# Unlocking Cholesterol Metabolism in Metabolic-Associated Steatotic Liver Disease: Molecular Targets and Natural Product Interventions

**DOI:** 10.3390/ph17081073

**Published:** 2024-08-15

**Authors:** Xiaoxiao Li, Meng Li

**Affiliations:** Institute of Digestive Diseases, Shanghai University of Traditional Chinese Medicine, Shanghai 200032, China; lxx767165719@163.com

**Keywords:** MASLD, MASH, cholesterol metabolism, natural products, hepatoprotection

## Abstract

Metabolic-associated steatotic liver disease (MASLD), the hepatic manifestation of metabolic syndrome, represents a growing global health concern. The intricate pathogenesis of MASLD, driven by genetic, metabolic, epigenetic, and environmental factors, leads to considerable clinical variability. Dysregulation of hepatic lipid metabolism, particularly cholesterol homeostasis, is a critical factor in the progression of MASLD and its more severe form, metabolic dysfunction-associated steatohepatitis (MASH). This review elucidates the multifaceted roles of cholesterol metabolism in MASLD, focusing on its absorption, transportation, biosynthesis, efflux, and conversion. We highlight recent advancements in understanding these processes and explore the therapeutic potential of natural products such as curcumin, berberine, and resveratrol in modulating cholesterol metabolism. By targeting key molecular pathways, these natural products offer promising strategies for MASLD management. Finally, this review also covers the clinical studies of natural products in MASLD, providing new insights for future research and clinical applications.

## 1. Introduction

Metabolic-associated steatotic liver disease (MASLD), formerly known as non-alcoholic fatty liver disease (NAFLD), has progressively been recognized as a predominant form of chronic liver disease, anticipated to ascend as the primary etiological contributor to end-stage liver diseases in subsequent decades. MASLD encompasses a range from simple hepatic steatosis to metabolic dysfunction-associated steatohepatitis (MASH), which can develop into advanced liver fibrosis, cirrhosis, and hepatocellular carcinoma (HCC). Corresponding with global amplification of obesity and type 2 diabetes mellitus (T2DM), epidemiological data suggests that MASLD affects an approximate 32.4% of the global populace [1]. Despite extensive research, the multifactorial etiopathogenesis of MASLD, involving insulin resistance, oxidative stress, pro-inflammatory cytokines, and lipotoxicity, remains incompletely understood [2]. A hallmark of MASLD is the dysregulated accumulation of neutral lipid species in the liver. Recent studies highlight the critical role of disturbances in cholesterol homeostasis in the pathogenesis and progression of MASLD.

Cholesterol plays a dual role in physiological processes, where both its deficiency and excess can lead to significant cellular and systemic issues [3]. Elevated hepatic free cholesterol (FC) has been observed in MASH patients compared to healthy individuals [4], with studies showing progressive increases in hepatic FC from non-MASLD to MASH stages [5]. The intrinsic role of cholesterol in hepatic steatosis and inflammation is underscored by experimental evidence linking high-fat-high-cholesterol (HFHC) diets to MASH development compared to high-fat-non-cholesterol regimens [6]. Furthermore, cholesterol-enrich ox-LDL uptake by hepatic Kupffer cells kindles inflammatory response in MASH [7], underscored by the lysosomal cholesterol crystal formation in macrophages, which serves as a danger signal activating inflammatory response [8]. Understanding the multifaceted roles of cholesterol in MASLD pathophysiology is crucial, as it could reveal novel molecular targets for therapeutic intervention.

Natural products, derived from diverse biological sources, encompassing fauna, flora, and microorganisms, have garnered attention for their purported protective attributes against MASLD. Several such compounds are posited to modulate hepatic cholesterol homeostasis, thereby substantiating their therapeutic potential in MASLD. Nonetheless, the mechanistic pathways through which these natural entities influence cholesterol processes remain to be exhaustively explored. This review aims to delineate the intricate processes of cholesterol absorption, transport, and metabolism under homeostatic conditions, provide a comprehensive overview of recent advances in cholesterol metabolic dysregulation in MASLD, and synthesize insights into the prospective therapeutic implications of natural compounds targeting cholesterol metabolic pathways for improving MASLD management.

## 2. Cholesterol Homeostasis in Health

### 2.1. Intestinal Cholesterol Absorption and Blood Release

Numerous dietary sources are rich in cholesterol, such as eggs, butter, seafood, and various meats. The American Cuisine Guidelines in 2015–2020 recommend a daily cholesterol intake not exceeding 300 mg [9,10]. The intestinal absorption of dietary cholesterol predominantly depends on the NPC1-like intracellular cholesterol transporter 1 (NPC1L1). This transporter, noted for its selective N-terminal domain, facilitates the endocytosis of FC into enterocytes, subsequently transporting it to the endoplasmic reticulum (ER) [11]. In the ER, FC undergoes esterification by acyl-CoA acyltransferase (ACAT), participating in the assembly of chylomicron (CM). These CMs are then secreted into the bloodstream via the lymphatic system [12]. In the peripheral blood, triglycerides in CMs are hydrolyzed by the lipoprotein lipase (LPL) on the vascular endothelium. CM remnants bind to the low-density lipoprotein receptor (LDLR) or the LDLR-related protein 1 (LRP) on hepatocyte membranes, facilitating their absorption by hepatocytes [13].

### 2.2. Reverse Cholesterol Transport (RCT)

RCT delineates the pathway wherein cholesterol from peripheral tissues is transported to the liver by high-density lipoprotein (HDL) and eventually excreted in feces. This mechanism is notably relevant to diseases such as MASLD, atherosclerosis (AS), and coronary heart disease, underscoring the protective function of HDL against elevated plasma lipids and AS. In peripheral serum, immature HDLs from the liver, consisting of phospholipids and apolipoprotein A-I (APOA-I), uptake FC released from adipocytes and muscles via the liver X receptor α (LXRα)-ATP-binding cassette transporter A1 (ABCA1)/ABCG1 pathways, maturing in the process. The maturation of HDL is mediated by lecithin cholesterol acyltransferase (LCAT), which converts cholesterol into cholesteryl esters (CEs) stored within HDL. Additionally, serum HDL levels are regulated by cholesteryl ester transfer protein (CETP), which transfers CEs from HDL to very-low-density lipoprotein (VLDL) and LDL [14]. Therefore, CETP and LCAT play pivotal roles in the RCT process. In the liver, HDL-c is selectively taken up, trafficked transhepatically, and excreted into bile via high-affinity HDL receptors such as scavenger receptor class B type I (SR-BI), thereby enhancing HDL metabolism, lowering plasma HDL-c levels, and promoting macrophage RCT.

### 2.3. Endogenous Cholesterol De Novo Synthesis

Apart from exogenous sources, cholesterol is synthesized endogenously within mammalian cells. This intricate biosynthetic process involves approximately 30 enzymatic reactions converting acetyl-CoA into cholesterol. Initially, two molecules of acetyl-CoA are condensed and catalyzed to generate mevalonate (MVA) by HMG-CoA synthase (HMGCS) and HMG-CoA reductase (HMGCR). MVA then undergoes a series of phosphorylation, decarboxylation, dehydroxylation, and condensation reactions to generate squalene, which is then catalyzed by ER cyclase and oxygenase to produce lanosterol, eventually yielding 27-carbon cholesterol [11,15]. Cholesterol de novo synthesis is mainly regulated by the sterol regulatory element-binding protein 2 (SREBP2). In low cholesterol conditions, the insulin-induced gene 1/2 (Insig1/2) dissociates from the SREBP cleavage-activating protein (SCAP) in the ER, prompting the SCAP–SREBP2 complex to transfer from the ER to the Golgi apparatus. Proteases S1P and S2P activate SREBP2, which enters the nucleus and activates target genes such as LDLR and HMGCR [16,17]. When cholesterol levels are excessive, cholesterol-derived oxysterols bind to Insig1/2, enhancing the affinity between Insig1/2 and SCAP and preventing SERBP2 transport towards the ER [18].

### 2.4. Cholesterol Conversion to Bile Acids (BAs)

**Figure 1:**
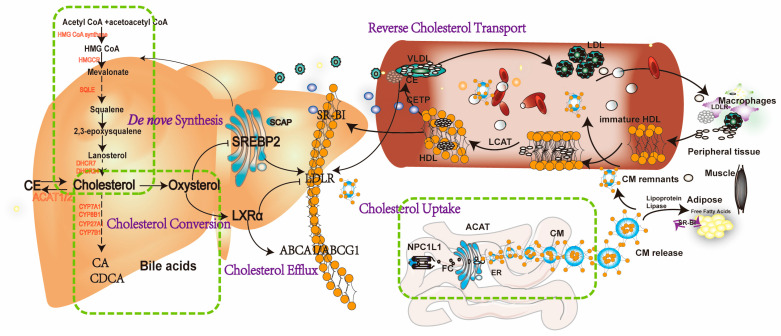
A schematic representation of cholesterol homeostasis in a host. The intestinal absorption of dietary cholesterol is phagocytosed into enterocytes, which depends on the NPL1L1. Subsequently, free cholesterol (FC) participates in assembling chylomicron (CM) by undergoing esterification via acyl-CoA acyltransferase (ACAT) and is secreted into the blood circulation. After hydrolyzed by the LPL in the circulating to diminish triglycerides, CM remnants are digested by low-density lipoprotein receptor (LDLR) or LDLR-related protein 1 (LRP) on the hepatocyte membrane, leading to their absorption by hepatocytes. In the peripheral serum, immature HDLs from the liver receive FCs from peripheral tissues and become mature by lecithin cholesterol acyltransferase (LCAT), which is responsible for converting cholesterol into cholesteryl esters (CEs) stored within HDL. Cholesteryl Ester Transfer Protein (CETP) transfers CEs from high-density lipoprotein (HDL) to very-low-density lipoprotein (VLDL) and LDL. In the liver, HDL-c are selectively uptake by scavenger receptor class B type I (SR-BI), thus resulting in improving HDL metabolism, lowering plasma HDL-c levels, and promoting macrophage reverse cholesterol transport (RCT). In the liver, cholesterol de novo biosynthesis involves approximately 30 enzymatic reactions, which convert acetyl CoA into cholesterol, such as HMG-CoA reductase (HMGCR), squalene epoxidase (SM), 24-dehydrocholesterol reductase (DHCR24) et al. Cholesterol homeostasis is mainly regulated by transcription factors sterol regulatory element-binding protein 2 (SREBP2) and liver X receptor α (LXRα). In low cholesterol conditions, the SREBP2 pathway activates and then enters the nucleus, activating the target gene such as LDLR and HMGCR. Once excessive cholesterol occurs, oxysterols can activate LXRα, promoting LXR-associated target genes, such as ABCA1 and ABCG1. Additionally, excess FCs are converted to CE stored in lipid droplets by ACAT1/2 or bile acids excreted into the bile duct by cholesterol 7 alpha-hydroxylase (CYP7A1), et al.

Cholesterol metabolism primarily occurs in hepatocytes. Remarkably, around 50% of intracellular cholesterol is metabolized into BAs, significantly contributing to the enterohepatic circulation, a key mechanism maintaining intracellular cholesterol homeostasis [19]. Evidence revealed that BAs are synthesized in hepatocytes via cytochrome P450 (CYP)-mediated oxidation of cholesterol, including both ‘classical’ and ‘alternative’ pathways. The ‘classical’ pathway, initiated by CYP7A1, progresses through various enzymatic stages involving CYP8B1 and CYP27A1, resulting in the formation of cholic acid (CA) and chenodeoxycholic acid (CDCA). The ‘alternative’ pathway generates CDCA by the synergistic enzymatic activities of CYP27A1 and CYP7B1 [20]. After their genesis, primary BAs are conjugated and transported from hepatocytes to the upper small intestine via the sphincter of Oddi, where they are extensively metabolized by bacterial enzymes. In the duodenum, BAs solubilize and enhance absorption of dietary lipids and lipid-soluble nutrients [21] (Figure 1).

## 3. The Role of Key Targets in Cholesterol Metabolism in MASLD

### 3.1. Cholesterol Absorption in MASLD

#### 3.1.1. NPC1L1

NPC1L1, a specific cholesterol uptake transporter, is predominantly located in the liver and small intestine. Studies indicate that NPC1L1 facilitates cholesterol absorption via vesicular endocytosis, dependent on microfilaments and the clathrin/AP2 complex [22]. Evidence suggests that dietary cholesterol significantly increases serum total cholesterol (TC) and LDL-c levels [23]. Inhibiting NPC1L1 with Ezetimibe reduces plasma LDL-c and apolipoprotein B (apoB)-containing lipoproteins by inhibiting the intestinal absorption of dietary cholesterol [24]. NPC1L1 knockout (NPC1L1^−/−^) mice manifested a substantial reduction in intestinal cholesterol uptake and a dramatical decrease in plasma phytosterol levels [25]. Conversely, overexpression of NPC1L1 in the liver results in a significant decrease in biliary cholesterol concentration and an increase in plasma cholesterol [26].

Numerous studies have reported a decline in cholesterol absorption and NPC1L1 expression in MASLD patients [27,28,29]. Recent studies identify hepatic NPC1L1 as a factor exacerbating MASLD. Elevated hepatic NPC1L1 exacerbates high-fat diet (HFD)-induced steatosis and is associated with a diminished hepatic capacity for VLDL-TG secretion [30]. Additionally, hepatic NPC1L1 inhibits hepatocyte autophagy, as indicated by decreased levels of microtubule-associated proteins 1A/1B light chain 3 (LC3) particles and the LC3II/LC3I ratio. Ezetimibe treatment can restore LC3II protein levels and improve liver steatosis [31]. Dietary oxysterols, such as 22(R)-hydroxycholesterol (22R-OHC) and 25-hydroxycholesterol (25-OHC), are believed to be associated with MASLD progression. NPC1L1 uptakes these oxysterols, which correlate positively with hepatic lipid accumulation in humans. Mechanically, these oxysterols contribute to steatosis progression through modulating LXRα and retinoid-related orphan receptor γ (RORγ) [32]. Oxidative stress is critical for MASLD pathogenesis, and the nuclear factor erythroid 2-related factor 2-Kelch-like ECH-associated protein 1 (Nrf2-Keap1) pathway is essential for providing cytoprotection against oxidative stress. Inhibiting NPC1L1 by ezetimibe activates the Nrf2-Keap1 pathway in a p62-dependent manner, decreasing active oxygen species (ROS) levels and hepatic susceptibility to oxidative damage. This underscores the potential antioxidant benefits of NPC1L1 inhibition in combating the pathogenesis of MASH [33]. Additionally, in a guinea pig model, ezetimibe effectively reduced diet-induced circulating cholesterol levels, hepatic lipid accumulation, and inflammatory response [34].

#### 3.1.2. LDLR

LDLR is a transmembrane protein primarily expressed in liver cells. It consists of a large extracellular domain with numerous ligand-binding domains and a cytoplasmic tail containing at least one NPxY motif. LDLR plays a crucial role in cholesterol homeostasis by clearing plasma cholesterol via binding circulating Apolipoprotein E (ApoE) and ApoB-containing particles such as chylomicrons, VLDL, and LDL. Approximately 70% of circulating LDL-c is eliminated via hepatic LDLR-mediated endocytosis. LDLR can be subjected to post-translational degradation by proprotein convertase subtilisin/kexin type 9 (PCSK9), a liver-derived solute factor that binds to the extracellular domain of LDLR, reducing LDL internalization and leading to hyperlipidemia. PCSK9 inhibitors like evolocumab and alirocumab are FDA-approved drugs for treating adult heterozygous familial hypercholesterolemia (FH) and clinical atherosclerotic cardiovascular diseases via inhibiting the binding of circulating PCSK9 and LDLR, enhancing the clearance of LDL-c from circulation. FH, an autosomal dominant disease caused by mutations in the LDLR gene, is characterized by high plasma LDL-c levels and accelerated MASLD and AS progression. Additionally, excessive cholesterol levels can activate LXRα, promoting LXR-associated target genes such as inducible degrader of LDLR (IDOL). LDLR can also be ubiquitinated and degraded by the E3 ubiquitin ligase IDOL, reducing intracellular cholesterol transport [35]. Thus, PCSK9 and IDOL serve as potential therapeutic targets for hyperlipidemia [36].

In MASLD, mice exhibit decreased hepatic LDLR and increased serum LDL-c levels [37]. Genetic ablation of LDLR in mice leads to liver injury, inflammatory cytokines releases, and high CD68 expression when fed a high cholesterol diet [38], as well as obesity and insulin resistance under a HFHC diet [39]. It is well-established that dietary cholesterol inhibits hepatic SREBP-mediated LDLR gene transcription, leading to reduced hepatic LDLR mRNA levels in hypercholesterolemic animals, which is a consequence of the feedback mechanism driven by high levels of cellular cholesterol in hepatocytes. However, other studies have shown that high cholesterol specifically accelerates LDLR mRNA degradation by inducing hepatic expression of LDLR mRNA decay-promoting factor heterogeneous nuclear ribonucleoprotein (HNRNP). Depletion of HNRNPD in the liver results in a marked reduction of serum LDL-c and a substantial increase in hepatic LDLR expression in hyperlipidemic mice [40]. In addition, LDLR expression is modulated by the epidermal growth factor receptor (EGFR)-extracellular signal-regulated kinase (ERK1/2) signaling pathway. Activating this pathway could improve MASLD by increasing LDLR in HepG2 cells [37].

Neutrophil infiltration around lipotoxic hepatocytes is a hallmark of MASH. Neutrophil-specific microRNA-223 (miR-223) in extracellular vesicles (EVs) can be preferentially taken up into hepatocytes to inhibit hepatic inflammatory and fibrogenic gene expression. This selective uptake depends on the expression of LDLR on hepatocytes and APOE on neutrophil-derived EVs. In the absence of this LDLR- and APOE-dependent uptake of miR-223-enriched EVs, the progression of steatosis to MASH is accelerated. Administration of the PCSK9 inhibitor Alirocumab enhances LDLR-dependent miR-223 transfer to ameliorate MASH in mice [41]. Although PCSK9 inhibition reduces plasma LDL-c and maintains LDLR expression in hepatocytes, it also increases liver exposure to cholesterol, potentially heightening the risk of MASH and HCC. PCSK9 knockout mice on a HFHC diet developed increased hepatic FC, cholesterol crystals, and fibrosing steatohepatitis, with a higher predisposition to liver cancer compared to wild-type mice. Future studies should evaluate whether patients on long-term anti-PSCK9 monoclonal antibody treatment are at increased risk of hepatic steatosis, MASH, or HCC, considering concurrent use of statins [42].

### 3.2. RCT in MASLD

#### 3.2.1. LCAT

LCAT is a lipid-modifying enzyme that catalyzes the transfer of the acyl chain from lecithin to the hydroxyl group of cholesterol on plasma lipoproteins, forming cholesteryl acylester and lysolecithin. Predominantly synthesized in the liver and secreted into plasma [43], LCAT plays a pivotal role in the RCT process. However, recent findings indicate that human LCAT overexpression substantially increases plasma HDL-c levels but does not enhance macrophage RCT, even with co-expression of SR-BI or CETP, which promote the transfer of LCAT-derived HDL cholesterol ester to the liver. LCAT-deficient mice show only a 50% reduction in RCT, and their serum promotes ABCA1-mediated cholesterol efflux from macrophages ex vivo. These data suggest that macrophage RCT may not be as dependent on LCAT activity as previously believed [44]. Additionally, LCAT deficiency often leads to reduced plasma HDL-c, corneal opacity, anemia, and renal issues. LCAT enzyme therapy might prevent serious complications, particularly renal dysfunction and corneal opacity [45].

Recent studies have associated higher LCAT activity with elevated fatty liver index (FLI) values, a surrogate marker of MASLD [46]. Specifically, subjects with an FLI ≥ 60 coinciding with type 2 diabetes mellitus (T2DM) and metabolic syndrome (MetS) exhibit an average 12% higher plasma LCAT activity. In age- and sex-adjusted partial linear regression analysis, LCAT activity is positively related to various obesity measures and homeostasis model assessment-insulin resistance (HOMA-IR). Multivariable linear regression analyses adjusted for age and sex show that LCAT activity is associated with an FLI ≥ 60, independent of T2DM and MetS, waist/hip ratio, or HOMA-IR. Therefore, MASLD inferred from an FLI ≥ 60 confers higher plasma LCAT and, to a lesser extent, phospholipid transfer protein activity, even when accounting for T2DM, MetS, central obesity, and insulin resistance [47]. In contrast, LCAT levels are found to be decreased in patients with MASLD and advanced fibrosis [48].

#### 3.2.2. CETP

CETP, predominantly originating from liver Kupffer cells, plays a pivotal role in lipoprotein metabolism by facilitating the transfer of esterified cholesterol from HDL to VLDL and LDL [14]. Clinical studies on CETP inhibitors such as torcetrapid, dalcetrapib, and evacetrapib aimed to curb AS by increasing HDL-c levels [49], though their expected efficacy remains unrealized, necessitating further research into the function of CETP [50,51,52,53]. Genetic CETP deficiency, mimicking pharmacologic CETP inhibition, is associated with a lower risk of cardiovascular morbidity and mortality but a markedly higher risk of age-related macular degeneration [54].

CETP gene expression is upregulated in MASLD induced by a high-cholesterol diet [55]. Patients with MetS with biopsy-proven MASLD (MetS+MASLD) show an increasing tendency of CETP activity. However, as MASLD progresses to severe fibrosis, CETP activity apparently improves [56]. Given that genetic polymorphism is an important factor in the pathogenesis of MASLD, a variant of the CETP gene polymorphism rs1800777 is independently associated with steatosis and lobulillar inflammation in subjects with biopsy-proven MASLD [57]. In addition, the B1B2/B2B2 genotype of CETP and elevated LDL-c serum levels increase the risk of MASLD in women with gallstone disease [58]. Considering that low HDL-c levels are associated with AS and MASH, increased HDL-c levels may reduce the risk of these diseases. A novel CETP vaccine (Fc-CETP6) efficiently elicited antibodies against CETP and reduced susceptibility to both AS and MASH induced by the HFHC diet via increasing plasma HDL-c and ApoA-I levels and decreasing plasma ox-LDL. Therefore, inhibition of CETP activity is considered a promising strategy for increasing HDL-c levels [59].

#### 3.2.3. SB-RI

SR-BI is a class B transmembrane scavenger receptor expressed in the liver, steroidogenic tissues, and adipose tissue [60]. SR-BI facilitates bidirectional cholesterol transport in vivo by the efflux of FC to HDL particles and the selective uptake of CE from HDL. SR-BI is required for insulin-mediated glucose uptake and regulation of energy balance in adipocytes. Loss of SR-BI in adipocytes resulted in inefficient glucose uptake compared to WT adipocytes, suggesting a novel role for SR-BI in glucose uptake and metabolic homeostasis in adipocytes [61]. Additionally, SR-B1 variants might influence adiposity markers in females [62]. SR-B1 deficiency regulates intestinal lipids, amino acids, and neurotransmitter metabolism in mice [63]. However, the crucial role of SR-BI in hepatocytes should also be noted. Research involving mice has shown that overexpression of hepatic SR-BI reduces plasma cholesterol levels but increases cholesterol excretion in feces. Conversely, SR-BI-deficient mice exhibited significantly increased plasma cholesterol with a corresponding decrease in fecal cholesterol excretion [64]. These findings demonstrate the paradoxical yet essential role of hepatic SR-BI. Although it inversely impacts steady-state plasma HDL-c concentrations, SR-BI emerges as an important positive regulator of hepatic RCT.

Generally, low HDL-c is a common feature of patients with MASLD. Hepatic SR-BI protein expression is lower in high-fat/high-sucrose (HFS) diet-induced mice [65]. Other studies report that gene and protein levels of hepatic SR-B1 in MASLD mice increase significantly compared to control mice. Furthermore, SR-B1 immunoreactivity increases and is mainly located in the plasma membrane of hepatocytes, cytoplasm, and the membrane of lipid droplets, with positive expressions associated with the severity of hepatic steatosis [66]. However, MASLD-relevant factors like inflammatory cytokines, lipopolysaccharide, and TGF-β do not affect SR-BI protein levels in primary human hepatocytes. Accordingly, hepatic SR-BI is not changed in human and murine hepatic steatosis and MASH. Therefore, the current study indicates a minor, if any, role of SR-BI in human and murine MASLD [67].

### 3.3. Cholesterol Biosynthesis in MASLD

#### HMGCR

HMGCR is a critical enzyme in cholesterol biosynthesis, localized to the ER. This enzyme catalyzes the conversion of 3-hydroxy-3-methylglutarylcoenzyme A (HMG-CoA) to mevalonate, a foundational precursor for cholesterol and other isoprenoids. HMGCR activity is primarily regulated through phosphorylation by AMP-activated protein kinase (AMPK), which inhibits the conversion of HMG-CoA to mevalonate, thereby reducing cholesterol synthesis [68,69]. In its dephosphorylated state, HMGCR is active, whereas phosphorylation by AMPK inhibits its activity. The degradation pathways of HMGCR are influenced by lanosterol, a seminal sterol intermediate in the mevalonate cascade, which facilitates the binding of INSIG to HMGCR [70]. This process triggers HMGCR ubiquitylation, extraction from the membrane, and proteasomal degradation via ER-associated degradation (ERAD) [11].

Empirical studies underscore a linkage between MASLD and perturbed HMGCR functionality. Elevated HMGCR expression, along with decreased phosphorylation of HMGCR, is a hallmark of MASLD and correlates with FC, histopathological severity of MASLD, and LDL-c levels [71]. In HFD-induced MASLD mice, hepatic TC and cholesterol biosynthesis genes such as SREBP2 and HMGCR levels are upregulated [72,73]. Western diet (WD) challenges further support these observations, with hepatic TC levels and HMGCR mRNA expression notably increased [74]. In vitro experiments using free fatty acid (FFA) or oleic acid (OA)-induced hepatic steatosis in HepG2 cells show surges in intracellular TG and TC, concomitant with increased HMGCR and SREBP2 levels [75,76]. Diving deeper into molecular mechanisms, HFDs have been shown to stimulate retinoic acid-inducible gene-I (RIG-I) expression, an RNA virus sensor in innate immune cells. RIG-1 amplifies cholesterol synthesis, precipitating steatosis and its sequelae, including MASH and hepatocarcinogenesis. Mechanistically, RIG-I undergoes constitutive methylation at K18 and K146 sites. In an intriguing twist, demethylase JMJD4 mediates RIG-I demethylation, consequently suppressing IL-6-STAT3 signaling. This methylated RIG-I form interacts with AMPKα, culminating in inhibiting HMGCR phosphorylation, thus promoting HMGCR’s enzymatic activity and fortifying cholesterol synthesis [77].

### 3.4. Cholesterol Efflux in MASLD

#### LXRα

LXRα, a pivotal member of the nuclear receptor superfamily, forms a dimer with retinoid X receptors, creating the RXR/LXR complex. This complex is modulated by rexinoids and oxysterol, such as 22(R)-hydroxycholesterol, 24(S)-hydroxycholesterol, and 24,25(S)-epoxycholesterol. Serving as a sophisticated sterol sensor, LXRα orchestrates a balance of sterol catabolism, storage, efflux, and elimination. For instance, LXRα regulates ABCA1, which is crucial for cholesterol absorption, leading to increased expression in jejunal enterocytes upon LXR agonist exposure. Furthermore, the ABCG5 and ABCG8 genes, under the regulation of LXR, enhance biliary cholesterol excretion [78]. Notably, a LXRα deficiency culminates in the liver’s inability to transcribe CYP7A, pivotal for cholesterol-to-BA conversion, leading to rapid hepatic cholesterol accumulation [79]. Consequently, liver-specific deletion of LXRα in mice impedes reverse cholesterol transport, catabolism, and excretion [80], emphasizing the paramount significance of LXRα in maintaining cholesterol homeostasis.

Accumulating evidence demonstrates the involvement of LXRα in MASH pathogenesis, particularly through the SREBP1c pathway [81]. MASLD patients exhibit increased expression of LXR and SREBP1c, with LXR overexpression closely linked to SREBP-1c augmentation [82]. Similarly, HFD-induced MASLD mice show increased LXRα mRNA expression [81,83], correlating with intrahepatic lipid accumulation and inflammation markers [28]. In both MASLD and hepatitis C virus (HCV)-afflicted patients with steatosis, hepatic LXRα expression, alongside lipogenic (e.g., PPAR-γ, SREBP-1c, SREBP-2, and FAS) and inflammatory genes (e.g., TNF-α, IL-6, and iNOS), is abnormally increased, pointing to the conceivable role of LXRα in these liver conditions’ pathogenesis [84]. Additionally, LXRα-knockout mice on HFHC diet demonstrated a surge in F4/80^+^CD68^+^CD11b^+^ macrophages, converging with hepatic cholesterol accumulation [85]. In conclusion, LXR expression reflects hepatic lipid deposition and hepatic inflammation in MASLD patients, highlighting LXR as a promising therapeutic target for hepatic inflammation and fibrosis [28].

### 3.5. Cholesterol Conversion in MASLD

#### CYP7A1

CYP7A1 stands at the biochemical crossroads of cholesterol metabolism, catalyzing the conversion of cholesterol into 7α-hydroxycholesterol, a pivotal precursor in BA synthesis. Deficiency in CYP7A1 in humans leads to significant metabolic aberrations, including impaired conversion of cholesterol to BAs, accumulation of hepatic cholesterol, reduction of LDL receptor expression, exacerbated hypercholesterolemia, and diminished statin response. Furthermore, this deficiency is associated with an increased risk of gallstone disease due to reduced bile acid secretion [86]. In CYP7A1^−/−^ mice, significantly elevated hepatic TC concentrations are observed compared to WT counterparts [87]. Conversely, upregulation of CYP7A1 can counteract cholesterol diet-induced hypercholesterolemia by preventing hepatic cholesterol accumulation and reducing the proliferation of apoB-containing lipoproteins in plasma, facilitated by augmented SREBP-mediated LDL receptor transcription [88].

In the context of MASLD, notable perturbations in BA metabolic profiles are evident, especially in individuals with MASH. These patients exhibit elevated total fecal and serum BAs, along with a reduced secondary-to-primary BA ratio compared to healthy individuals [89,90]. Within the MASLD/MASH spectrum, increased CYP7A1 expression is commonly observed, particularly when compared to control cohorts [91]. Therapeutically, modulating BA synthesis by downregulating CYP7A1 presents a potential approach to mitigate MASLD progression [92,93]. However, divergent data indicates a marked suppression of CYP7A1 in HFD-induced MASLD [72]. This discrepancy highlights the complex interplay between the farnesoid X receptor (FXR) signaling pathway and MASLD-associated BA dysregulation. FXR exerts an inhibitory effect on CYP7A1 transcription, mediated through the intestinal fibroblast growth factor 19 (FGF19)/FGFR4/β-Klotho axis or the hepatic small heterodimer partner (SHP). A therapeutic strategy that involves counteracting FXR signaling while upregulating CYP7A1 may offer a promising remedy for MASLD [94,95]. Additionally, the cholesterol metabolic enzyme 25-hydroxylase (Ch25H) and its product 25-hydroxycholesterol (25-HC) offer another therapeutic proposition. Their role in alleviating HFD-induced hepatic steatosis, mediated via the LXRα–CYP7A1 axis, suggests a novel strategy to address the multifaceted challenges of MASLD [96].

## 4. Natural Product Targeting Cholesterol Metabolism for MASLD

**Figure 2:**
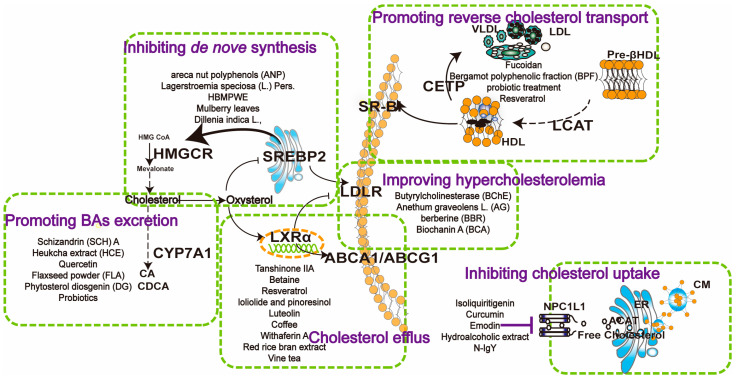
Mechanisms of natural products in regulating cholesterol metabolism in MASLD. NPC1L1 inhibitors such as isoliquiritigenin, curcumin, and emodin reduce intestinal cholesterol uptake. Enhancing reverse cholesterol transport by compounds such as fucoidan, bergamot polyphenolic fraction, and probiotics, which enhance CETP, SR-B1, and LCAT expression, facilitating cholesterol transfer from peripheral tissues to the liver. Inhibiting de novo synthesis involves compounds like areca nut polyphenols, *Lagerstroemia speciosa*, and HBMPWE, mulberry leaves, which suppress HMGCR activity, reducing cholesterol synthesis. Promoting bile acid excretion through Schizandrin A, Heuchka extract, and quercetin upregulates CYP7A1, enhancing cholesterol conversion to bile acids. Butyrylcholinesterase, *Anethum graveolens* L., and berberine improve hypercholesterolemia by lowering cholesterol levels at various metabolic points. Cholesterol efflux is promoted by compounds like tanshinone IIA, betaine, and resveratrol, which enhance ABC transporter activity (ABCA1/ABCG1), promoting cholesterol removal from cells to HDL. These pathways collectively maintain cholesterol homeostasis, offering potential therapeutic strategies for MASLD.

As mentioned above, the dysregulation of cholesterol metabolism and key targets is a critical factor in the progression of MASLD. Despite the lack of approved specific therapeutic drugs, numerous natural products have exhibited therapeutic potential for MASLD in vitro and in vivo. Herein, we summarize the hepatoprotective effects of natural products targeting crucial cholesterol metabolic pathways (Figure 2), including inhibiting cholesterol absorption (NPL1L1 and LDLR), enhancing RCT, inhibiting cholesterol biosynthesis, modulating the LXRα pathway, and promoting BA excretion via CYP7A1.

### 4.1. Inhibiting Cholesterol Absorption

#### 4.1.1. NPCL1L Inhibitors

Recent studies have demonstrated that various natural products can reduce the cellular uptake of cholesterol by inhibiting the NPC1L1 protein. For example, isoliquiritigenin, a flavonoid with a chalcone structure extracted from the natural herb *glycyrrhiza glabra*, has been shown to downregulate NPC1L1 expression and competitively inhibit cellular cholesterol uptake by binding to NPC1L1 in a concentration-dependent manner in vitro [97]. A systematic review and meta-analysis evaluating the therapeutic efficacy of oral dietary polyphenols in patients with MASLD indicated that curcumin significantly decreases AST, ALT, TG, TC, and HOMA-IR compared to placebo [98]. In vitro, curcumin inhibits cholesterol uptake in Caco-2 cells by down-regulating NPC1L1 expression [99]. Additionally, in vivo supplementation with curcumin reduces intestinal cholesterol absorption and improves HFD-induced dyslipidemia by downregulating the expression of NPC1L1 [100].

Emodin, an active component of the traditional Chinese medicine rhubarb, also demonstrates cholesterol-lowering properties. Mechanically, studies showed that NBD-cholesterol uptake in human HepG2 cells decreased significantly after treatment with various concentrations of emodin, compared to control and ezetimibe treatments. Thus, emodin inhibited cholesterol uptake by HepG2 and Caco-2 cells more effectively than ezetimibe. This inhibition occurs via NPC1L1 in an anti-competitive manner [101]. The hydroalcoholic extract obtained from the açai seed (ASE), can reduce NPC1L1 and increase the expression of ABCG5 and PPAR-α to improve lipid profile and attenuate hepatic steatosis in HFD-induced MASLD mice [102]. Moreover, N-IgY, a chicken egg yolk-derived IgY specific for NPC1L1, has been shown to block cholesterol transport efficiently in HepG2 and Caco-2 cells, with an effect comparable to ezetimibe. N-IgY combined with omega-3 polyunsaturated fatty acids led to significant upregulation of genes involved in cholesterol uptake (LDLR), reverse cholesterol transport (ABCG5/ABCG8), and BA metabolism (CYP7A1), representing a promising treatment strategy to prevent HFD-induced MASLD through the activation of cholesterol catabolism to BAs and by decreasing cholesterol-induced fibrosis [103].

#### 4.1.2. LDLR Upregulation

The ERK signaling cascade is a central regulator of LDLR expression in MASLD. Caffeine markedly improved HFD-induced MASLD by activating EGFR-ERK1/2 signaling and promoting LDLR expression in ApoE KO mice [37]. Liver butyrylcholinesterase (BChE) governs LDL-c levels and LDL uptake capacity through the MEK-ERK signaling cascade to promote LDLR transcription. These findings suggest that targeting liver LDLR is an effective therapeutic strategy to treat MASLD and hypercholesterolemia [104]. *Anethum graveolens* L. (AG), an annual plant known for its hypolipidemic effects, has shown promise in normalizing liver histopathological changes in high-cholesterol diet-fed animals by significantly increasing liver antioxidants and upregulating LDLR expression. This modulation leads to improved circulating cholesterol and LDL-c clearance [105]. Berberine (BBR) has effectively ameliorated MASLD via lowering glucose levels, reducing inflammatory factors, and inhibiting endotoxin release. Furthermore, BBR administration could reverse the abnormal expression of MTTP and LDLR to improve HFD-induced MASLD. Mechanistically, berberine stabilizes LDLR mRNA and increases LDLR expression, leading to increased cholesterol catabolism [106]. Additional evidence indicates that the stabilization of LDLR mRNA induced by berberine is dependent on ERK activation [107]. Biochanin A (BCA), an ox-methylated isoflavone found in soybeans, red clover, alfalfa, peanuts, and chickpeas, delays liver damage of MAFLD, though significantly increasing CYP7A1, LDLR, and PPAR-α protein expression while downregulating HMGCR, SREBP-1c, and PPAR-γ protein expression. This regulation promotes cholesterol absorption and metabolism, thereby protecting against MASLD progression [72].

### 4.2. Enhancing RCT

RCT is a physiological process where excess peripheral cholesterol is transported to the liver for excretion into the bile and subsequently the feces. Fucoidan, derived from *Ascophyllum nodosum*, has been shown to lower lipid levels by modulating RCT-related protein expression. Specifically, fucoidan enhances lipid transfer from plasma to the liver by activating SR-B1 and LDLR while inactivating PCSK9. It also upregulates lipid metabolism by activating ABCA1, ABCG8, and CYP7A1 [108]. Accumulating evidence indicates that bergamot polyphenolic fraction (BPF) can improve histopathological and serum biomarkers of MASLD. This suggests a correlation between BPF and improved biochemical modulation in liver tissues. BPF restores the dysregulated lipid transfer protein system via normalizing serum concentrations of lipemic biomarkers and the activity of ACAT, LCAT, and CETP. This normalization leads to a decrease in hepatic cholesterol content and improved lipoprotein trafficking in the liver, contributing to the hypolipemic response observed in MASLD patients [109]. Probiotic treatment has also been found to significantly protect against MASLD by restoring gut flora, improving liver functions, and reducing inflammatory cytokines. For example, *Lactobacillus acidophilus* supplementation can normalize the expression of lipid-related genes affected by a high-cholesterol diet. This is achieved by upregulating CETP, lipoprotein lipase (LPL), and hepatic lipase (HL), while downregulating LDLR [55]. Resveratrol has been shown to protect against HFHS-induced decreases in hepatic LDLR and SR-BI gene and protein expressions, providing new insights into its pharmacological targets in MASLD prevention [65]. Considering hypercholesterolemia as a leading cause of MASLD, many natural products can attenuate hypercholesterolemia by upregulating LCAT and SR-BI expression and downregulating CEPT expression, thereby accelerating RCT. Examples include curcuminoids, *Desmodium gyrans* methanolic extract, a 5% ethanol extract of *unripe Rubus coreanus*, and the ethanol extract of *Edgeworthia gardneri* (Wall.) Meisn [110,111,112,113].

### 4.3. Inhibiting Cholesterol Biosynthesis

Inhibiting cholesterol biosynthesis is a key strategy in managing MASLD, as it helps reduce hepatic cholesterol levels and prevent lipid accumulation. Areca nut polyphenols (ANP), derived from the areca nut, have been found to reduce WD-induced TC and non-high-density lipoprotein (non-HDL) levels by increasing the abundance of beneficial bacteria in the gut microbiota and reducing the expressions of SREBP2 and HMGCR. The mechanism involves increasing the expression of phosphorylated AMPKα, which inhibits cholesterol synthesis in MASLD mice [114]. DLBS3733, a bioactive fraction of *Lagerstroemia speciosa* (L.) Pers., shows potential in treating hepatic steatosis-related diseases through downregulating the expression of HMGCR and SREBP and upregulating CPT-1 in HepG2 cells. SREBP directly activates the expression of genes involved in cholesterol synthesis, and its downregulation by DLBS3733 helps repress hepatic steatosis [115]. In high-fat, high-fructose, high-cholesterol diet (HFFCD)-fed MASLD hamsters, treatments with HBMPWE—a product fermented by inoculating *Monascus purpureus* on highland barley fruit—significantly inhibited lipid accumulation. This was achieved by downregulating proteins related to fatty acid synthesis and cholesterol synthesis (SREBP-1/ACC/FAS/AceS1 and HMGCR) and upregulating cholesterol clearance (CYP7A1) [116]. Mulberry leaves (MLF) exhibit regulatory effects on abnormal cholesterol metabolism, improving hepatic injury in MASLD. In vivo studies show that MLF treatment significantly downregulates the hepatic expression levels of SREBP2, HMGCR, and miR-33a. In vitro, quercetin, an active metabolite of MLF, significantly decreases lipid accumulation in HepG2 cells by inhibiting the gene and protein expression level of SREBP2 and HMGCR [117]. *Dillenia indica* L., an edible plant from the Dilleniaceae family present in India and other Asian countries, significantly reduced the expression of SREBP-1c, SREBP-2, HMGCR, FAS, and CD36 in oleic acid-treated cells, thereby alleviating lipid accumulation [76].

### 4.4. Modulating LXRα Pathways

Abnormal and excessive accumulation of lipid droplets within hepatic cells is the main feature of MASLD. The LXRα–SREBP1 pathway plays a crucial role in hepatic steatosis and the pathological progression of MASLD. Tanshinone IIA, a bioactive phytochemical from *Salvia miltiorrhiza* Bunge, attenuates lipid accumulation by modulating the LXRα–SREBP1 pathway to treat MASLD [118]. Loliolide and pinoresinol, identified in the dichloromethane fraction, significantly accelerate the protein degradation of LXRs by enhanced ubiquitination. This reduces the expression levels of lipogenic factors including SREBP-1, SCD1, FAS, and ACC, thus inhibiting lipogenesis and improving MASLD [119]. Luteolin can abolish lipid accumulation induced by LXR-SREBP-1c activation both in vivo and in vitro, indicating its potential as a therapeutic agent for treating MASLD [120]. Additionally, betaine prevents MASLD by reversing the expressions of LXRα and PPARα in the liver and promoting the expression of genes related to fatty acid oxidation [121].

As a dual LXR/FXR receptor activator, withaferin A activates both LXR-α and FXR, inducing their canonical target genes (ABCA1 and ABCB11) and inhibiting diet-induced hepatic steatosis, steatohepatitis, and fibrosis [122]. Red rice bran extract (RRBE) attenuates markers of inflammation such as NF-κB and iNOS genes in the liver and alleviates the expression of key genes involved in cholesterol metabolism. Specifically, RRBE decreases CD36 and HMGCR levels while increasing LXRα expression in HFD-induced MASH, demonstrating its anti-inflammatory properties [123]. Emerging evidence suggests AMPK-LXRα participates in the development of MASH [124]. AMPK activators have also been shown to inactivate LXRα. Chinese vine tea (VTE) alleviated the progression of MASH via enhancing AMPK and blocking LXRα signaling in mouse livers [125]. Diosgenin is another potential agent for preventing the development of MASLD through the AMPK and LXR signaling pathways [126]. Current studies have identified that coffee intake reduces HFD-induced liver macrovesicular steatosis and serum cholesterol levels. Coffee supplementation prevents HFD-induced MASLD in mice by modulating liver LXRα expressions. LXRα regulates systemic cholesterol homoeostasis by increasing biliary cholesterol excretion through the regulation of the intestinal membrane transporters ABCA1 and ABCGl, thereby impacting intestinal cholesterol efflux and improving MASLD [127]. Combining caffeine with chlorogenic acid exerts collaborative effects in HFD-fed mice via the AMPKα–LXRα/SREBP-1c pathway [128]. Resveratrol and atorvastatin administration elevated ABCA1 and ABCG1 and reduced LXRα protein expression to improve MASLD by targeting genes involved in cholesterol metabolism [129].

### 4.5. Promoting BA Excretion via CYP7A1

CYP7A1 is a rate-limiting enzyme for the conversion of cholesterol to BAs. Enhancing CYP7A1 expression through natural products has shown potential in improving MASLD by promoting bile acid excretion. Schizandrin A, a lignan found in the fruits of the Schisandra genus, significantly alleviates HFHC diet-induced MASLD. This is achieved by markedly increasing the expression of CYP7A1 and ABCA1, facilitating biliary cholesterol excretion and cholesterol efflux to HDL in the liver [130]. Heukcha extract (HCE), a naturally post-fermented green tea extract, has been shown to suppress diet-induced MASLD. This effect is achieved by significantly increasing the protein level of CYP7A1 in the liver [131]. Quercetin, known for its hepatoprotective effect on T2DM-associated MASLD, down-regulates nuclear YY1, which directly binds to the CYP7A1 promoter and activates its transcription. This results in the restoration of cholesterol homeostasis via the conversion of cholesterol to BAs [132].

Previous studies show that BA imbalance in MASLD is closely associated with the FXR signaling pathway. FXR negatively regulates CYP7A1 transcription by fibroblast growth factor 19 (FGF19)/FGF4/klothoβ in the intestine or by small heterodimer partner (SHP) in the liver. Phytosterol diosgenin (DG) notably decreases hepatic cholesterol through increasing hepatic CYP7A1 and prohibiting FXR-mediated signaling, thus contributing to cholesterol elimination and alleviating HFD-induced hypercholesterolemia [94]. Flaxseed powder (FLA) is rich in α-linolenic acid, dietary fiber, lignans, and other active ingredients. Animal experiments reveal that FLA intervention significantly activates the intestinal FXR-FGFR4-CYP7A1 to lower lipid and modulate TGR5-TLR4-TNFα pathways to alleviate inflammation in HFD-induced MASLD [133]. Probiotics are prospective for the prevention and treatment of hypercholesterolemia and MASLD. For example, *L. plantarum* WLPL21 showed the best mitigatory effect on HCD-induced hypercholesterolemia, including upregulating cholesterol metabolism (CYP27A1, CYP7B1, CYP7A1, and CYP8B1) levels in the liver, cholesterol transportation (ABCA1, ABCG5, and ABCG8) in the ileum or liver, and downregulating NPC1L1 [134] (Table 1).

## 5. Clinical Trials of Natural Products for the Treatment of MASLD

The promising preclinical efficacy of natural products targeting cholesterol metabolism for MASLD has led to their clinical evaluation. Although only a few of the 25 natural products mentioned have been tested in clinical trials for MASLD, the results are encouraging (Table 2). (1) Curcumin: Systematic review and meta-analysis show curcumin reduces liver enzymes (AST and ALT), body weight, waist circumference, body fat percentage, and body mass index (BMI) compared to placebo [136,137,138]. A randomized double-blind placebo-controlled trial (80 mg/day for 3 months) in overweight/obese MASLD patients showed improvements in various metabolic parameters, indicating anti-inflammation, antidiabetic, and lipid-lowering effects [139]. Another trial (500 mg/day for 8 weeks) confirmed benefits, though some patients experienced stomachache and nausea, leading to three drop-outs [140]. (2) Berberine: A meta-analysis of 10 randomized controlled trials involving 811 patients demonstrated its efficacy in improving liver function, serum lipid profile, and insulin sensitivity in MASLD patients [141]. Treatment with Berberis integerrima extract (750 mg twice/day for 2 months) significantly decreased BMI, serum lipid profile, fasting blood glucose (FBG), and liver enzymes, while increasing HDL-c, glutathione peroxidase enzyme, and total antioxidant capacity [142]. Another trial found berberine (0.5 g three times/day for 16 weeks) more effective than pioglitazone (15 mg/day) in reducing body weight and improving serum lipid profile, despite some digestive side effects [143]. (3) Resveratrol: Clinical trials on the effects of resveratrol on liver enzymes in MASLD are mixed [144]. Five trials involving 216 patients showed significant changes in ALT and AST levels [145]. However, a randomized, double-blind, placebo-controlled trial (600 mg/day for 12 weeks) showed significant reductions in body weight, BMI, and waist circumference [146]. Another trial found resveratrol (500 mg/day for 12 weeks) with lifestyle modification superior to lifestyle modification alone in reducing inflammation and hepatocellular apoptosis [147]. Further studies are needed to confirm the effect of resveratrol on liver enzymes. (4) Quercetin: A randomized clinical trial combining rosuvastatin with quercetin (40 mg three times/day for 3 months) showed significant reductions in oxidative stress, enhanced antioxidant protection activity, and decreased hepatocyte apoptosis [148]. (5) Hesperidin and flaxseed: In a randomized, controlled clinical trial, hesperidin (1 g/day) and flaxseed (30 g/day) for 12 weeks improved glucose and lipid metabolism while reducing inflammation and hepatic steatosis in MASLD patients, showing synergistic effects on FBG and HOMA-IR [149].

Despite these promising results, most other natural products have been evaluated primarily in animal models rather than clinical trials, necessitating further validation. Adequate subject numbers and balanced sex ratios are essential for objective results in clinical trials. Existing human clinical trials often focus on simple biomarkers, lacking a comprehensive multi-indicator evaluation system associated with the functional targets of natural products.

## 6. Concluding Remarks and Future Perspectives

MASLD represents a significant global health issue, being the leading cause of hepatogenic mortality and affecting approximately one-fourth of the global population. Previous studies have identified that various natural products can mitigate MASLD by reducing intestinal cholesterol absorption and hepatic cholesterol levels. This review emphasizes the hepatoprotective effects of natural products targeting crucial metabolic pathways, including uptake (NPL1L1 and LDLR), transport (CETP, LCAT, and SRBI), biosynthesis (HMGCR), efflux (LXRα), and conversion (CYP7A1). Despite these advancements, several challenges remain in utilizing natural molecules for MASLD therapy. Many natural compounds in the biological world remain undiscovered. With the rapid development of bioinformatics and artificial intelligence, the discovery of more efficient and low-toxicity natural products is anticipated. These advancements could address poor clinical efficacy and side effects associated with some natural molecule drugs. Moreover, small molecules often exhibit poor water solubility and low oral availability, necessitating further experiments to better understand their absorption and conversion in vivo. Additionally, the complex and dynamic nature of cholesterol-related metabolic homeostasis requires further exploration. These limitations pose significant challenges in developing and practically applying natural therapeutic molecules for treating MASLD. A deeper understanding of how natural products modulate cholesterol homeostasis is fundamentally important for establishing novel therapeutic approaches to improve MASLD.

## Figures and Tables

**Table 1:** Natural products targeting cholesterol dysfunction in MASLD.

Natural Products	Source	Interfering Mechanism	Molecular Target	Models	Refs
Isoliquiritigenin	*Glycyrrhiza glabra*	Cholesterol absorption	NPC1L1	HepG2 Caco-2 cells	[97]
Curcumin	Turmeric curcuminoids	Cholesterol absorption	NPC1L1	Caco-2 ApoE^−/−^ mice	[100]
Emodin	Rhubarb, Cassia, and Heshouwu	Cholesterol absorption	NPC1L1	HepG2 Caco-2 cells	[101]
Hydroalcoholic extract	*Euterpe oleracea* Mart.	Cholesterol absorption Cholesterol excretion	NPC1L1 ABCG5	C57BL/6 mice	[102]
N-IgY	A chicken egg yolk-derived IgY	Cholesterol uptake RCT Cholesterol conversion	NPC1L1 ABCG5 ABCG8 CYP7A1 LDLR	HepG2 Caco-2 cells C57BL/6	[103]
Leaf extracts of Anethum graveolens	*Anethum graveolens*	Improve hypercholesterolemic	LDLR	Hamsters	[105]
Berberine	Coptidis Rhizoma	Improve lipid	LDLR	Sprague-Dawley rats	[106]
Biochanin A	Soybeans, Red clover, Alfalfa, peanuts, and chickpeas	Cholesterol uptake RCT Cholesterol conversion	CYP7A1 LDLR HMGCR	Sprague-Dawley rats	[72]
Fucoidan A2	Brown seaweed Ascophyllum nodosum	RCT Cholesterol conversion Cholesterol efflux	ABCA1 ABCG8 LDLR SR-BI CYP7A1	C57BL/6J mice	[108]
Bergamot polyphenolic fraction	Bergamot	RCT	LCAT CETP	Male Wistar rats	[109]
*Lactobacillus acidophilus*	Probiotics	RCT	CETP LDLR	Rabbits	[55]
Resveratrol	Polygonum cuspidatum	RCT Cholesterol efflux	LDLR SRBI LXRα ABCA1 ABCG1	Wistar male rats	[65,129]
Schizandrin A	Schisandra genus	Cholesterol excretion Cholesterol efflux	ABCA1 CYP7A1	C57BL/6J mice	[130]
Heukcha	Green tea extract	Bile acid synthesis	CYP7A1	Golden Syrian hamsters	[131]
Quercetin	Flavonoid	Cholesterol conversion	CYP7A1	HepG2 cells Mice	[132]
Flaxseed powder	Alpha linolenic acid	Cholesterol conversion	CYP7A1	C57BL/6 mice	[133]
*Lactiplantibacillus plantarum WLPL21*	Probiotics	Cholesterol transportation Cholesterol conversion	NPC1L1 CYP27A1 CYP7A1 ABCA1 ABCG5 ABCG8	C57BL/6 mice	[134]
*Lactobacillus fermentum* CQPC06	Fermented pickles	Cholesterol conversion	CYP7A1	C57/BL6J mice	[135]
Coffee	Coffee beans	Cholesterol efflux	LXRα ABCA1	C57BL/6J mice	[127]
Diosgenin	yams	Cholesterol absorption RCT Cholesterol conversion	SRBI CYP7A1 LXRα	Rat	[94,126]
Areca nut polyphenols	Areca nut	Cholesterol synthesis	SREBP2 HMGCR	C57BL/6N mice	[114]
DLBS3733	*Lagerstroemia speciosa (L.) Pers.,*	Cholesterol synthesis	HMGCR ACC SREBP	HepG2 cells	[115]
HBMPWE	*Monascus purpureus*	Cholesterol synthesis Cholesterol conversion	CYP7A1 HMGCR	Golden hamsters,	[116]
*Morus alba L.,* flavonoids	Mulberry leaves	Cholesterol synthesis	CYP7A1 SREBP2 HMGCR	Rats	[117]
Hydroethanolic extract of *Dillenia indica* L.,	*Dillenia indica* L.	Cholesterol synthesis	SREBP2 HMGCR	HepG2 cells	[76]

**Table 2:** Clinical trial efficacy and safety of natural products on MASLD.

Natural Products	Administration and Dose	Outcomes	Clinical Study Type	Clinical Trial Registration	Adverse Effects (AE)	Refs
Berberine	12 weeks, 1500 mg/day	Significant decrease in serum ALT, serum TC, and de Ritis ratio	Randomized, double-blind placebo-controlled trial	NCT05523024	Mild and transient gastrointestinal reactions	[150]
Berberine	16 weeks, 0.5 g three times/day	Improved body weight, HOMA-IR, and serum lipid profiles	Randomized parallel controlled open-label trial	NCT00633282	Anorexia and upset stomach, diarrhea, and constipation	[143]
Berberis integerrima extract	2 months, 750 mg twice/day	Decreased BMI, serum lipid profiles, FBG, liver enzymes, and renal parameters; increased serum HDL-c, glutathione peroxidase enzyme, and total antioxidant capacity	Randomized clinical trial	IR.RUMS.REC.1396.110.	None	[142]
Curcumin	12 weeks, 250 mg/day	Reduced steatosis and fibrosis with FibroScan; reduced waist circumference and blood pressure	Double-blind parallel placebo-controlled trial	IRCT20121216011763N39	No adverse events	[151]
Curcumin	60 days, 160 mg/day	Decreased serum ALT and AST	Double-blind randomized clinical trial	IRCT2017012031423N1	None	[152]
Curcumin	8 weeks, 250 mg/day	Reduced ALT, AST, body weight, waist circumference, body fat percentage, and BMI	Randomized, double-blind controlled trial	IRCT2015052322381N1	No severe adverse effects	[153]
Curcumin	2 months, 500 mg/day	Reduced ALP concentrations and severity of MASLD	Double-blind placebo-controlled trial	IRCT2015052322381N1	None	[154]
Curcumin	12 weeks, 1500 mg/day	Reduced hepatic fibrosis, serum cholesterol, FBG, and ALT	Randomized placebo-controlled clinical trial	None	None	[155]
Curcumin	12 weeks, 500 mg/day	Reduced serum ALT, ALP, TC, LDL-c, iron, and hemoglobin; increased total iron-binding capacity	Randomized controlled parallel-group trial	UMIN000033774	None	[156]
Curcumin	3 months, 80 mg/day	Improved fatty liver degree, liver transaminases, waist circumference, FBG, FBI, HbA1c, TG, TC, LDL, HOMA-IR, TNF-α, hs-CRP, and IL-6	Double-blind randomized placebo-controlled trial	IRCT2016071915536N3	Nausea	[139]
Curcumin	8 weeks, 500 mg/day	Reduced liver fat content, BMI, TC, LDL-c, AST, ALT, FBG, and glycated hemoglobin	Randomized double-blind placebo-controlled trial	IRCT2014110511763N18	Stomachache and nausea	[140]
Caffeine	12 weeks, 200 mg/day	Reduced body weight and energy intake	Randomized, placebo-controlled trial	NCT02929901	None	[157]
Flaxseed powder	12 weeks, 30 g/day	Decreased BMI, glucose hemostasis parameters, and hepatic steatosis	Randomized parallel controlled open-label trial	NCT03734510	No adverse effects reported	[149]
Resveratrol	12 weeks, 600 mg/day	Reduced body weight, BMI, and waist circumference	Randomized double-blind placebo-controlled trial	IRCT201511233664N16	No adverse events	[146]
Resveratrol	6 months, 50 mg/day or 200 mg/day	Reduced liver fat, liver enzymes, serum glutamate pyruvic transaminase, gamma-glutamyl transpeptidase, and insulin resistance	Randomized clinical trial	ΕΕΒΚ/ΕΠ/2010/12	None	[158]
Resveratrol	12 weeks, 500 mg/day	Reduced serum ALT, inflammatory cytokines, nuclear factor κB activity, serum cytokeratin-18, and hepatic steatosis grade	Randomized double-blinded controlled trial	NCT0203097	No significant adverse effects	[147]

## Abbreviations

ACAT	Acyl-CoA acyltransferase
ALP	Alkaline phosphatase
ALT	Alanine aminotransferase
AMPK	AMP-activated protein kinase
APOA-I	Apolipoprotein A-I
AST	Aspartate aminotransferase
BMI	Body mass index
CA	Cholic acid
CDCA	Chenodeoxycholic acid
CE	Cholesteryl ester
CETP	Cholesteryl ester transfer protein
CM	Chylomicron
CYP7A1	Cholesterol 7 alpha-hydroxylase
ER	Endoplasmic reticulum
ERK	Extracellular regulated protein kinase
Ev	Extracellular vesicle
FBG	Fasting blood glucose
FBI	Fasting blood insulin
FC	Free cholesterol
FH	Familial hypercholesterolemia
FFA	Free fatty acid
HCV	Hepatitis C virus
HCC	Hepatocellular carcinoma
HDL	High-density lipoprotein
HFD	High-fat diet
HOMA-IR	Homeostasis model assessment-insulin resistance
HMG-CoA	3-hydroxy-3-methylglutarylcoenzyme A
Insig1/2	Insulin-induced gene 1/2
LCAT	Lecithin cholesterol acyltransferase
LDLR	Low-density lipoprotein receptor
LPL	Lipoprotein lipase
LXRα	Liver X receptor α
MASLD	Metabolic-associated steatotic liver disease
MASH	Metabolic dysfunction-associated steatohepatitis
MetS	Metabolic syndrome
RIG-I	Retinoic acid-inducible gene-I
PCSK9	Proprotein convertase subtilisin/kexin type 9
RCT	Reverse cholesterol transport
SCAP	SREBP cleavage-activating protein
SHP	Small heterodimer partner
SR-BI	Scavenger receptor class B type I
SREBP2	Sterol regulatory element-binding protein 2
TC	Total cholesterol
T2DM	Type 2 diabetes mellitus
VLDL	Very-low-density lipoprotein
WD	Western diet
